# Chick early amniotic fluid component improves heart function and protects against inflammation after myocardial infarction in mice

**DOI:** 10.3389/fcvm.2022.1042852

**Published:** 2022-11-16

**Authors:** Juan Wang, Xiejiu Chen, Lihong Zhang, Yufan Zheng, Jin Qian, Ning Sun, Xiaolei Ding, Baiping Cui

**Affiliations:** ^1^School of Medicine, Institute of Geriatrics (Shanghai University), Affiliated Nantong Hospital of Shanghai University (The Sixth People’s Hospital of Nantong), Shanghai University, Nantong, China; ^2^Shanghai Engineering Research Center of Organ Repair, School of Medicine, Shanghai University, Shanghai, China; ^3^State Key Laboratory of Medical Neurobiology, Department of Physiology and Pathophysiology, School of Basic Medical Sciences, Fudan University, Shanghai, China; ^4^Cancer Institute (Key Laboratory of Cancer Prevention and Intervention, China National Ministry of Education), The Second Affiliated Hospital, Zhejiang University School of Medicine, Hangzhou, China; ^5^Zhejiang HygeianCells BioMedical Co., Ltd., Hangzhou, China; ^6^Stem Cell Application Research Center, Hangzhou Branch of Yangtze Delta Region Institute of Tsinghua University, Hangzhou, China; ^7^Wuxi School of Medicine, Jiangnan University, Wuxi, China

**Keywords:** amniotic fluid, myocardial infarction, hESC-derived cardiomyocytes, inflammation, immune cells

## Abstract

Myocardial infarction (MI) is the major cause of mortality around the world. We recently demonstrated that chick early amniotic fluid (ceAF) can effectively rescue ischemic heart injury, indicating that it has a therapeutic function in MI. However, its functional components and the underlying mechanisms remain to be clarified. Here, we demonstrated that a fraction of ceAF, peak 8 (P8), had a protective effect on acute MI. P8 significantly decreased cardiomyocyte cross-sectional areas and cardiomyocyte apoptosis in MI mice. Using a human embryonic stem cell-derived cardiomyocyte model, which was subjected to hypoxia and reoxygenation, mimicking MI state, we found that P8 treatment reduced apoptosis and reversed myocardial contractility. Mechanistically, P8 improved cardiac function by inhibiting NF-κB signaling and downregulating inflammatory cytokine expression. Using mass spectrometry, we identified that guanosine and deoxynucleoside were the main functional components of P8 that suppressed the inflammatory response in human embryonic stem cell-derived cardiomyocytes. Collectively, our data suggest that specific components from ceAF are promising therapeutic agents for ischemic heart injury and could be a potential supplement to current medications for MI.

## Introduction

Ischemic heart disease, such as acute myocardial infarction (MI), is a major reason of morbidity and mortality in the world (1). As a common result of coronary artery disease, MI induces a large loss of cardiac cells, resulting in cardiac dysfunction and even heart failure (1). Ischemia-induced myocardial cell death following MI contributes to progressive heart remodeling (2). A set of events, including infiltration and activation of inflammatory cells, proliferation of resident cardiac fibroblasts, and remodeling of extracellular matrix accompanied with scar formation, dynamically proceed, eventually leading to left ventricular (LV) remodeling toward dilatation and hypertrophy (3, 4). Particularly, immune responses at early phase are associated with cardiac remodeling after injury (5, 6). Growing evidence indicates that timely shortening of the acute inflammatory period of MI can attenuate LV dysfunction, and heart failure, and ensure optimal heart repair. It has been shown that targeting circulating CCR2^+^ monocytes/macrophages or loss of CCR2 can reduce inflammation in the heart after ischemic-reperfusion (I/R) (6–8), and *Ccr2* deficiency notably improves cardiac function after I/R (7). In addition, anti-inflammatory CX3CR1^+^ macrophage accumulation rejuvenates the function of I/R-injured hearts after cardiac adult stem cell therapy (7).

The NF-κB signaling pathway plays an important role during inflammatory responses by regulating inflammatory cytokine expressions (9). After MI, activation of NF-κB promotes inflammatory responses, which brings out LV remodeling and deterioration of function (10). Hypoxia induced pro-inflammatory interleukin (IL) -6 and CCL2 (MCP-1) in right and left ventricles, and upregulated NF-κB (11), leading to ventricle remodeling. Inhibiting NF-κB can reduce myocardial remodeling and dysfunction following MI (12–15).

Amniotic fluid (AF) is important for fetal development by forming a protective sac around the fetus. AF maintains acid/base balance and contains nutrients, growth factors, and cytokines (16, 17). As a supplement of natural medium, 6- and 10- day-old chick embryo AF can sustain the development of two-cell mouse embryos (18). Moreover, it has been shown that chick embryo AF can promote peripheral nerve regeneration in rats (19) and rat AF can prevent intraperitoneal adhesion formation in surgical operation (20). A more recent study showed that human AF (hAF) reduces C-reactive protein, a marker of inflammation, and improved clinical outcomes in hospitalized patients with COVID-19 after intravenous 10 ml hAF without obvious adverse events (21), indicating an immune-mediating role of hAF. Previously, we demonstrated that chick early amniotic fluid (ceAF) can effectively rescue heart dysfunction after ischemic injury in swine and mouse models (22). ceAF contains multiple fractions and the functional components of cardio-protective effect remain to be explored. In this study, we have investigated the main active components of ceAF and its potential mechanisms on cardio-protection effects.

## Materials and methods

### Animals

All C57BL/6 mice and SD rats used in this research were 8–10 weeks old. Monkeys (Macaca fascicularis) were purchased from Guangxi Xiongsen Primate Experimental Animal Co., Ltd. (Guangxi, China). Animal experiment protocols and ethics were approved by the Institutional Animal Care and Use Committee (IACUC) of Shanghai University, Fudan University, or the Suzhou JOINN Laboratories.

During the surgical procedure and echocardiography, mice were anesthetized by inhaling isoflurane. To harvest hearts for *ex vivo* experiments, mice were sacrificed by excessive inhalation of isoflurane. Rats were anesthetized with isoflurane and euthanized by abdominal aorta bloodletting. Monkeys were euthanized by CO_2_ inhalation.

### Preparation and separation of P8

Preparation and separation of ceAF from 7-days chick embryo were performed as previously described (22). Briefly, the eggs impregnated with male seed were incubated at ∼38°C with 50% humidity for 7 days (23). AF extract liquid was centrifuged at 12,000 r/min for 30 min and the supernatants were filtered and collected for fresh use or storing at-80°C for long-term use. The fractions of peak 1–10 were separated according to the peak time of HPLC. Briefly, Acetonitrile (ACN) and ddH_2_O were used as mobile phases and separated the different fractions by C^18^ reverse phase separation column. The first thing is to equilibrate reversed phase column until the UV absorption curve at 280 nm was stable and returned to the baseline with mobile phase 3% ACN. Loading was at the 1 ml/min flow rate and gradient elution was at the 10 ml/min flow rate. The fraction is collected in equal volume, separated and purified repeatedly for 20 times. Combine each fraction with the same peak time.

### Acute myocardial infarction model

Establishment of MI mouse model was performed as previous procedures (24). Briefly, under sterile conditions, anesthetized mice received a left intercostal thoracotomy allowing heart exposure and the left anterior descending (LAD) was ligated by a 7–0 silk suture permanently, which caused the anterior wall and apex to become blanching. The Sham mice model experienced the same operation without ligating the anterior descending branch of left coronary artery.

### Human embryotic stem cells culture and directed differentiation

Human embryonic stem cells (hESCs), line H7, were cultured in serum-free mTeSR1 medium (Stemcell Technologies, #85852) on Matrigel-coated culture plates and passaged when cells reached suitable confluence.

Cardiomyocyte differentiation was carried out when cells reached 85–95% confluence. For differentiation, cells were cultured with RPMI/B-27 medium. Gsk3 inhibitor CHIR-99021(12 mM, Selleck, S2924) or a WNT inhibitor IWR-1 (5 mM, Sigma, I0161) was added to the cells on day 0 and 2–3, respectively. On day 4–6, cells were treated with fresh RPMI/B-27 medium. Contracting cells appeared between 7 and 9 days post differentiation and the hESC-derived cardiomyocytes after ≥ 25 days differentiation were used in the experiments. The successful differentiation of myocardial cells can be determined by the appearance of beating cells, as the beating cells and three-dimensional cell bulge can be observed in the plates when the induced differentiation goes well.

### Echocardiography

Assessment of cardiac function used the Vevo 2100 micro ultrasound system (Visual Sonics, Canada). Maintained mild anesthesia with gas anesthesia, Cross section of heart was imaged in two-dimensional long-axis view and M mode cursor at the level of the greatest LV diameter. Echocardiography was performed on week 1, 2, 3, and 4 after treatment. LVEF, LVFS, LVIDD and LVIDS were calculated at all time-points.

### Toxicological study

Toxicological studies were conducted by daily intravenous infusion of ceAF P8 fraction for 1 week in healthy adult SD rats or monkeys by JOINN Laboratories. Body weight, body temperature, heart rate and electrocardiogram were measured before and after P8 administration. Serum and urine were collected before and after P8 administration for biochemical detection.

### Fibrotic scar area quantification

Mouse heart tissues were harvested, embedded in paraffin. Sectioned tissues were processed for Masson’s trichrome staining. Image J was used to quantize the blue fibrotic areas on trichrome.

### Immunohistochemistry

Tissues were harvested, washed with cold PBS buffer and fixed for either paraffin embedding or OCT embedding. H&E, Masson’s trichrome and LY-6G immunohistochemistry (IHC, 1:200, #87048s, Cell Signaling Technology, US) staining were carried out. For frozen tissue sections, after permeabilization and blocking, tissue slides were probed with the following primary antibodies: CX3CR1 (1:200, ab8201, Abcam, US), CCR2 (1:200, ab203128, Abcam, US), MPO (1:200, ab208670, Abcam, US). After washes with PBS, samples were processed with fluorescent secondary antibodies for 1 h, followed by DAPI staining for 10 min to visualize nucleus. Images were visualized with fluorescence microscope (Carl Zeiss, Germany).

### Western blot

RIPA buffer containing protease inhibitors was used to tissue lysis and protein extraction. Equal amount of protein was separated by SDS- PAGE and transferred to PVDF membranes. After non-specific protein blocking treatment, PVDF membranes were incubated with the specific primary antibodies overnight at 4°C. The 60-min incubation of secondary antibodies was carried out after TBST washing. Immunoblots were imaged using chemiluminescence and did quantitative analysis by the Image J software. The antibodies used as follows: Bax (1:1000, ##2772, CST, US), Bcl2 (ab196495, Abcam, US), Caspase 3 (#9662, CST, US), GAPDH (# 5174, CST, US).

### Flow cytometry analysis

Single cell suspension was obtained from mouse spleen. Briefly, tissue was lysed with ammonium-chloride-potassium (eBioscience) and filtered to remove red blood cells. Following treatment with blocking antibody to restrain non-specific binding, suspended cells were incubated with fluorochrome-conjugated antibodies for 30 min at 4°C. Flow cytometry analysis was conducted using FACS Calibur (BD Biosciences) and data were processed using Flow Jo software (TreeStar, Inc.). CD3 antibody (Clone17A2, BioLegend), CD4 antibody (Clone RM4.5, BD Biosciences) and CD8 antibody (BD Biosciences) were used in this study.

### Enzyme-linked immunosorbent assay

To effectively produce P8-specific antibodies, one group of mice were subcutaneously injected with 50 μl P8 accompanied with 10 ng LPS (lipopolysaccharides) per mouse. For P8-specific antibody measurement, serum was collected at day 8 and day 22 after tail vein injection of P8. Enzyme-linked immunosorbent assay (ELISA) plates (Costar) were pre-coated with 100 μl P8 at 4°C for 18 h. Rabbit anti-mouse IgG (Abcam) and rabbit monoclonal (RM109) to mouse IgM (Abcam) conjugated with HRP were used. Read the board at 450 nm wavelength (Tecan) after incubation with HRP substrate TMB (Abcam).

### Ribonucleic acid extraction and sequencing

Total ribonucleic acid (RNA) extract, cDNA libraries establishment and sequencing analysis were performed according to previous method (24).

### Quantitative real-time polymerase chain reaction

Same equivalent RNA (-1 μg) was reverse transcribed into cDNA and Real-time polymerase chain reaction (PCR) was performed according the instructions. The primer sequences for RT-qPCR were listed in Supplementary Tables 1, 2.

### Statistical analysis

Analytical charts were made by GraphPad Prism 8. The data were presented as mean ± SEM. *t*-test was used for comparisons of the differences between two groups. One-way ANOVA was used for comparing differences among more than two groups. ns ≥ 0.05 was considered as no statistical significance, and *p < 0.05 and **p < 0.01 ***p < 0.001, ****p < 0.0001. were considered as statistical significance.

## Results

### ceAF fraction P8 reduces cardiomyocyte apoptosis and improves cardiac function in MI mice

Our previous study indicated that ceAF can effectively rescue heart function after acute myocardial ischemic injury by intravenous administration in mice and swine (22). However, the active component was still unclear. AF was isolated from chick embryo (Supplementary Figure 1A) and presented 10 peaks on high performance liquid chromatography (HPLC) chromatogram (Supplementary Figure 1B). Compared with days 8–13 AF, day 7 chick embryo AF was safe without significant effect on survival rate, body weight, serum glucose levels and organ histological morphology after 1-week daily intravenous infusion initially followed 39 weeks weekly intravenous injection in healthy adult C57BL/6J mice (Supplementary Figure 2). However, days 8–10 chick embryo AF containing more protein (25) increased body weight, and intravenous injection of days 11–13 AF sharply decreased the survival rate of mice after 40 weeks (Supplementary Figure 2). Thus, ceAF referred to AF on day 7, was used in subsequent experiments.

**FIGURE 1:**
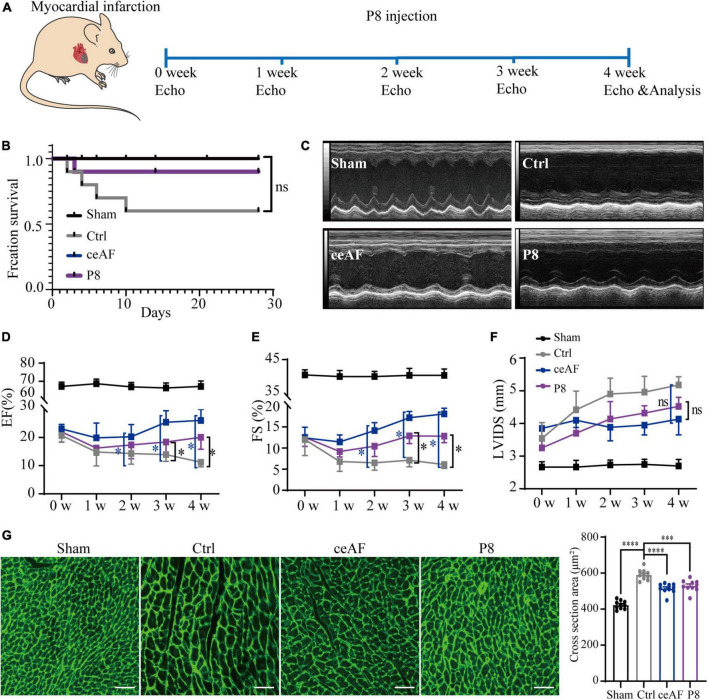
P8 application improves heart function of MI mice. **(A)** Scheme illustrating schedule of P8 treatment after MI and echocardiography analysis. **(B)** Survival curve of Sham, untreated (control) and ceAF and P8 treated mice after MI. *n* = 10 per group. **(C)** Representative echocardiography at 4 weeks after MI. Quantitative evaluation of cardiac contractile function **(D,E)** and cardiac remodeling **(F)** with 5% glucose (MI control), ceAF and P8 treated mice after MI and sham control mice. The groups of experimental mice are: sham, *i.v.* injection of 5% glucose (Ctrl), *i.v.* injection of 5.0 ml/kg of ceAF (ceAF), injection P8 fraction 5.0 ml/kg (P8). *n* = 6 per group. **(G)** Representing WGA staining of cardiac cross sectional area post-MI (*n* = 10 per group). Scale bar: 50 μm. *p < 0.05; ***p < 0.001; ****p < 0.0001; ns, not significant.

To further explore the main active components of ceAF, we separated ceAF by HPLC and 10 fractions (P1-P10) were collected according to the peak time (Supplementary Figures 1A,B). After tail intravenous injection of Peak 1–10 in MI mice, we found that P8 treatment showed a strong therapeutic effect for improving heart function compared to other fractions (Supplementary Figures 1C,D). To further substantiate a direct role of P8 in improving cardiac function post-MI, mice were administrated with P8 (5.0 ml/kg, dissolved in 5% glucose) for 4 weeks, and heart function were measured weekly through echocardiography with histochemical analysis at end time (Figure 1A). P8 treatment reduced the postoperative death of MI mice (Figure 1B) and increased ejection fractions (EF), fractional shortening (FS) demonstrated improved cardiac function. Left ventricular internal systolic diameter (LVIDS) was decreased, although the numbers were statistically insignificant (Figures 1C–F) in comparison with control in post-MI mice. Moreover, apoptotic cardiomyocytes were replaced by fibroblasts after MI and the fibrotic tissue don’t have the function of myocardial tissue, leading to ventricular dilation and enlargement of myocardial cells in the border zone of infarction. Wheat germ agglutinin (WGA) staining revealed that the myocardial ultrastructure was recovered and cross-sectional area was reduced in the P8 group post-MI (Figure 1G).

**FIGURE 2:**
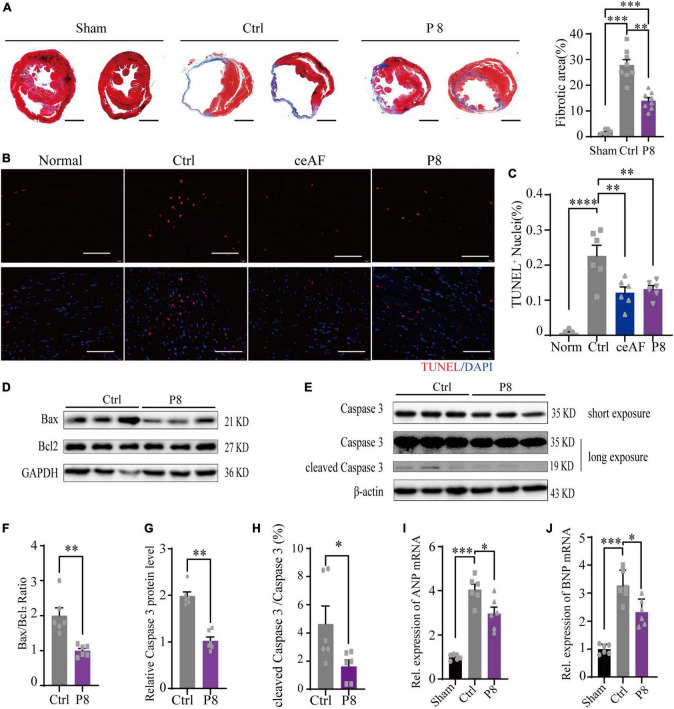
P8 ameliorates myocardial injury in MI mice. **(A)** Representative images of heart Masson’s trichrome staining from unchallenged (sham), MI mice (control), and P8 treated MI mice (P8) (4 weeks). (Right) the statistical analysis of fibrotic area relative to myocardium in trichrome staining sections, *n* = 8 per group. Scale bar: 2μm. **(B)** TUNEL staining of heart tissue sections in the border zone of infarction 6 h after MI. **(C)** Percent of TUNEL^+^ nuclei in mouse heart sections after 6 h with or without P8 treatment (*n* = 6 per group). Scale bar: 100 μm. **(D–H)** P8 decreased cardiac Bax/Bcl2, Caspase3 protein expression and cleaved Caspase 3/Caspase 3 ratio post-MI (*n* = 6 per group). P8 reduced cardiac ANP **(I)** and BNP **(J)** mRNA expression post-MI (*n* = 6 per group). *p < 0.05; **p < 0.01; ***p < 0.001; ****p < 0.0001.

Cardiac fibrosis area reflects the severity of heart failure and is negatively correlated with cardiac function after MI in mice. Masson staining showed that P8 administration significantly reduced collagen deposition in the infarction zone of post-MI heart, indicating less cardiac fibrosis (Figure 2A). Cardiomyocyte apoptosis is the fundamental driver of ischemic heart injury. TUNEL assay revealed that apoptotic cells at the border zone of infarction in P8 group were significantly reduced compared to the control (Figures 2B,C), indicating that P8 fraction has anti-apoptosis effects in MI heart. Consistently, both Bax/Bcl2 ratio and cleaved Caspase 3 protein levels were decreased after P8 administration (Figures 2D–H). Additionally, the transcripts of atrial natriuretic peptide (ANP) and brain natriuretic peptide (BNP), two markers for heart failure, were increased in MI and were decreased post P8 treatment (Figures 2I,J). Collectively, these data indicate that P8 can improve cardiac function post-MI with a similar protective effect as ceAF.

### P8 protects hESC-derived cardiomyocytes from the harmful effects caused by hypoxia/reoxygenation injury

**FIGURE 3:**
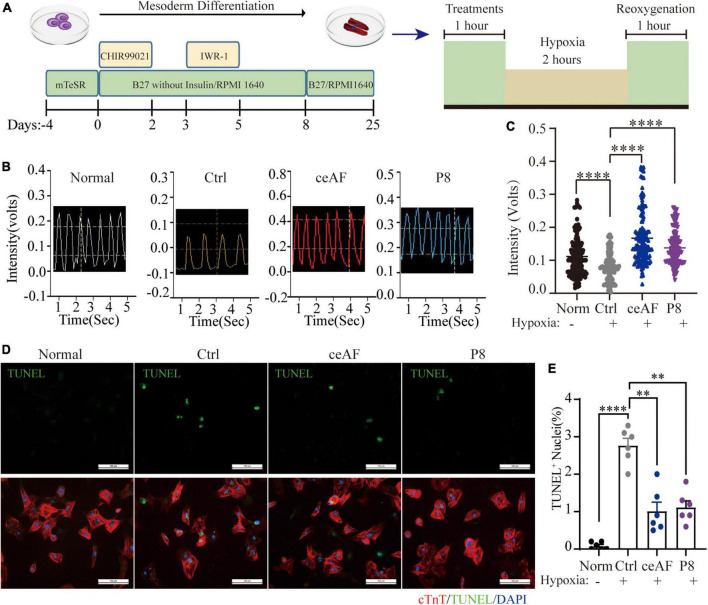
P8 protects hESC-derived cardiomyocytes from the harmful effects of hypoxia/reoxygenation. **(A)** The schematic protocol used for hESC differentiation into cardiomyocytes. Differentiated cardiomyocytes were pretreated with or without P8 for 1 h, hESC-derived CMs were suffered either normoxia or hypoxia in a hypoxic cell incubator (1 O_2,_ 5 CO_2_, and 94% N_2_) for 2 h followed by 1 h of reoxygenation. **(B)** Representative traces of contraction force recording of hESC-derived CMs pretreated with 20% ceAF, 20% P8 or DMEM and hypoxia/reoxygenation process. *n* = 6 per group. **(C)** Quantification of relative contraction force of hESC-derived cardiomyocytes treated with 20% ceAF, 20% P8 or DMEM and hypoxia/reoxygenation process. Each point in the figure represents the contractility of a single cardiomyocyte. **(D)** TUNEL and TNNT2 staining of hESC-derived cardiomyocytes suffered either normoxia or hypoxia in a hypoxic cell incubator (1 O2, 5 CO2, and 94% N2) for 2 h followed by 1 h of reoxygenation (*n* = 6 per group). **(E)** Percent TUNEL^+^ nuclei in hESC-derived cardiomyocytes after hypoxia/reoxygenation process with or without P8 treatment (*n* = 6 per group). Scale bar: 100 μm. **p < 0.01; ****p < 0.0001.

The contracting inducible cardiomyocytes can mimic human cardiomyocytes and have been used to assess beating state (26, 27). To assess whether P8 has a conserved function on human cardiomyocytes, we next established a hypoxia/reoxygenation hungry (2% FBS) model of human cardiomyocytes, mimicking the ischemia-reperfusion state of cardiomyocytes (Figure 3A), and the hypoxia/reoxygenation cell model includes hypoxic injury similar with MI model and reoxygenation injury. Human ES cells were differentiated into cardiomyocytes using a differentiation method as previously described (28) (Figure 3A). Myocardial cells began to contract at days 8–10 of differentiation and the cardiomyocytes at around day 25 of differentiation were used for further analyses (Figure 3A). Hypoxia/reoxygenation treatment resulted in the decreased pace and amplitude of hESC-derived cardiomyocytes. Similar to MI mouse model, P8 pretreatment protected the cells from the harmful effects (Figures 3B,C). P8 reduced the TUNEL^+^ human cardiomyocytes after hypoxia/reoxygenation injury (Figures 3D,E), validating its conserved role in anti-apoptosis. Overall, these data indicated that P8 protects cardiomyocytes from hypoxia/reoxygenation effects, possibly through inhibiting apoptosis.

### P8 fraction does not show significant toxicity and immunogenicity

Having gained functional insights of P8 function in MI mice and hESC-derived cardiomyocytes with hypoxia/reoxygenation effects, we next investigated its toxicity and immunogenicity with three models, including mice, rats and monkeys. Daily intravenous infusion of P8 was performed in healthy adult SD rats (6–8 weeks old) and monkeys (*Macaca fascicularis*) (2–6 years old) for 1 week. Blood biochemistry, T lymphocyte subsets, and cytokines were tested after 7 days of P8 infusion (Supplementary Figure 3A). Three experimental groups (SD rats) were infused daily with 1.5 ml/kg, 5.0 ml/kg, and 15.0 ml/kg of P8, respectively, and no obvious changes were observed in the blood cell counts (Supplementary Figures 3B,D). Moreover, food intake and body weight gain were comparable in all groups (Supplementary Figure 3C). Similarly, incremental dosages of 1.5 ml/kg, 5.0 ml/kg, and 15.0 ml/kg P8 were daily infused into monkeys. There were no noticeable side effects of P8 from multiple test outcomes, including body weight, body temperature, heart rate, electrocardiogram, blood cell counts, coagulation function, blood biochemistry, urine analysis, T lymphocyte subsets, cytokines, and immunoglobulin (Supplementary Figures 3E–J). The IL-6 levels in all three treatment groups showed a possible tendency to increase in a dose- depended manner with an outlier in the highest dosage treated group (Supplementary Figure 3H), but there was no statistical difference.

**FIGURE 4:**
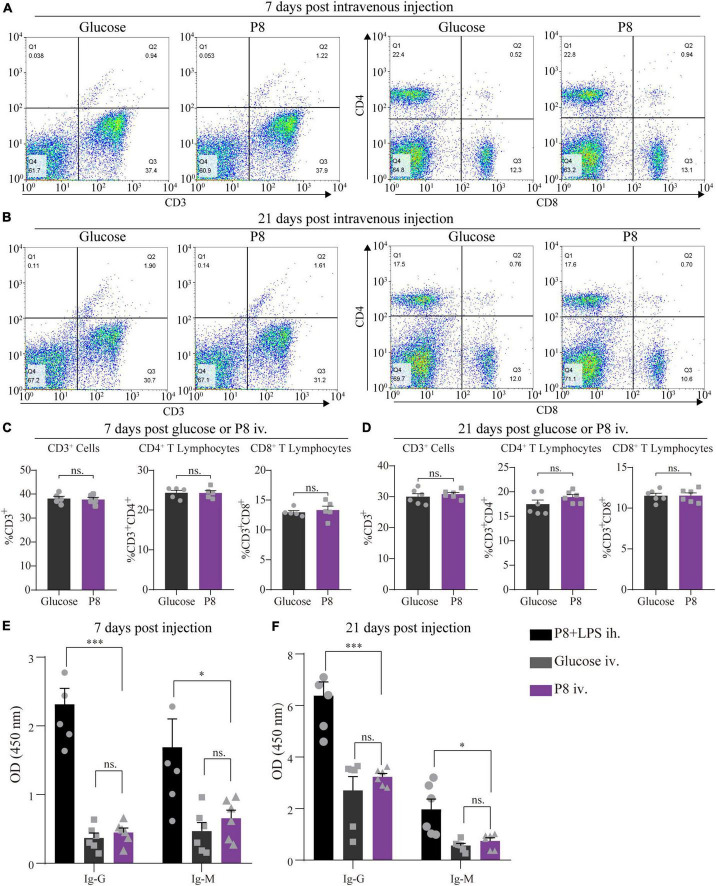
No significant P8-specific antibody production and CD4^+^, CD8^+^ T lymphocytes response after P8 intravenous injection. **(A,B)** The percentage of splenic CD4^+^ and CD8^+^ T cells were detected by flow cytometry after 7 days injection daily and 21 days intravenous injection of P8 (7 days injection daily following 14 days injection every other day of P8). **(C,D)** Statistical analysis of Flow cytometry detection. Compared with the control, flow cytometric analysis showed that animals with intravenous injection of P8 for 7 days **(C)** and 21 days **(D)** presented no significant change of CD4^+^, CD8^+^ T lymphocytes. *n* = 6 per group. P8-specific IgG and IgM levels were detected in mouse sera by ELISA after 7-day **(E)** and 21-day **(F)** P8 injection. P8 + LPS ih., P8 + 10ng LPS, hypodermic injection (positive control group); Glucose iv, 5% glucose, intravenous injection; P8 iv, P8, intravenous injection. *n* = 6 per group. *p < 0.05; ***p < 0.001.

To further assess the potential immunogenicity of P8, 8-week-old mice received daily injection with P8 for the first week and every other day for the next 2 weeks. Spleens and serum were harvested at day 7 or day 21 after the first injection. No significant changes were observed in the numbers of splenic CD3^+^, CD3^+^CD4^+^, and CD3^+^CD8^+^ lymphocytes after repeated P8 administration (Figures 4A–D). There was also no significant antibody production after repeated P8 administration (Figures 4E,F) through ELISA test of Ig G and Ig M. These data suggest that P8 did not induce any observable immunogenicity in mice.

### P8 downregulates NF-κB signaling pathway in cardiomyocytes

**FIGURE 5:**
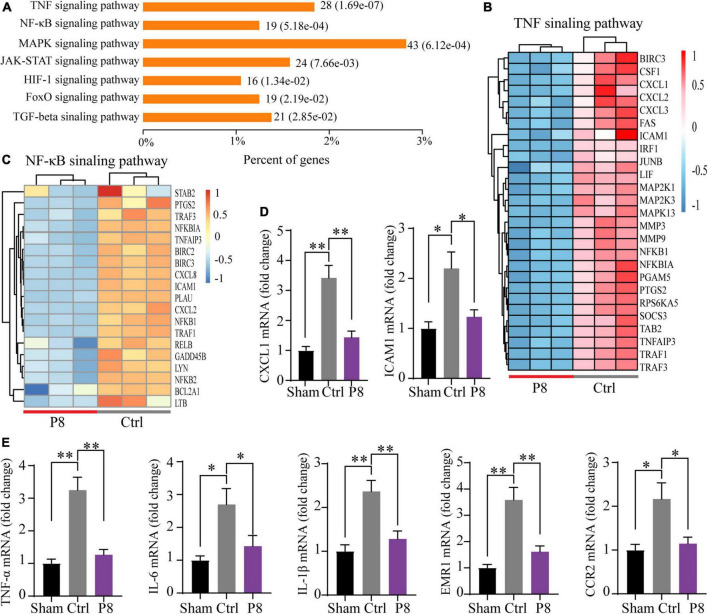
P8 attenuates inflammatory response in hESC-derived cardiomyocytes subjected to hypoxia/reoxygenation injury. **(A)** KEGG pathway enrichment analysis of differentially expressed genes between in the P8-treated group vs. the Ctrl group of hESC-derived cardiomyocytes subjected to hypoxia in a hypoxic cell incubator for 2 h followed by 1 h of reoxygenation (*n* = 3 per group). **(B)** Heatmap shows the selected differentially expressed transcripts (normalization with Z-score, adjusted *p*-value < 0.05) of TNF signaling pathway. **(C)** Heatmap shows the differentially expressed transcripts (normalization with Z-score, adjusted *p*-value < 0.05) of NF-κB signaling pathway. **(D,E)** Real-time PCR analysis of the expressions of **(D)** inflammation-related genes (CXCL-1, ICAM1) and **(E)** proinflammatory molecules (TNF-α, IL-6, IL-1β, EMR-1 and CCR1) in hESC-derived cardiomyocytes after hypoxia-reoxygenation treatment (*n* = 3) cultures. *p < 0.05; **p < 0.01.

To get insight into the underlying molecular mechanism of the protective effects from P8 administration, we performed whole genome RNA sequencing analyses comparing transcriptomes of hESC-derived cardiomyocytes experienced to hypoxia for 2 h followed by reoxygenation for 1 h with or without P8 treatment. Compared with the control (treated with 5% glucose), there were 4,108 genes upregulated and 2,361 genes downregulated in hESC-derived cardiomyocytes after P8 treatment (Supplementary Figure 4A). Through KEGG pathway analysis, we identified that TNF-α-NF-κB signaling pathway was most significantly changed in down-regulated genes (Figure 5A), such as chemokines *CXCL1*, *CXCL2*, *CXCL3*, *CXCL8*, and inflammation-related genes *ICAM-1*, *NF*κ*B1*, *NF*κ*B2* (Figures 5B,C). Gene set enrichment analysis (GSEA) of transcriptomes confirmed a consistent set of down-regulated genes associated with TNF and NF-κB signaling pathways (Supplementary Figure 4B). To validate these observations, we performed real-time qPCR. Consistent with RNA-Seq results, P8 downregulated *CXCL1*, *ICAM1*, *TNF*-α,*IL6*, *IL-1*β, *EMR1* and *CCR2* transcription (Figures 5D,E). These data imply a working mechanism of P8 modulation of NF-κB signaling pathway induced by hypoxia injury. Moreover, the expression of apoptosis- and hypertrophic cardiomyopathy (HCM)- related genes was also significantly downregulated (Supplementary Figures 4C,D), which was in line with the results of anti-apoptosis and reducing cardiomyocyte cross-sectional area in the P8 group (Figures 2B–I).

### P8 inhibits NF-κB signaling pathway after myocardial injury in mice

**FIGURE 6:**
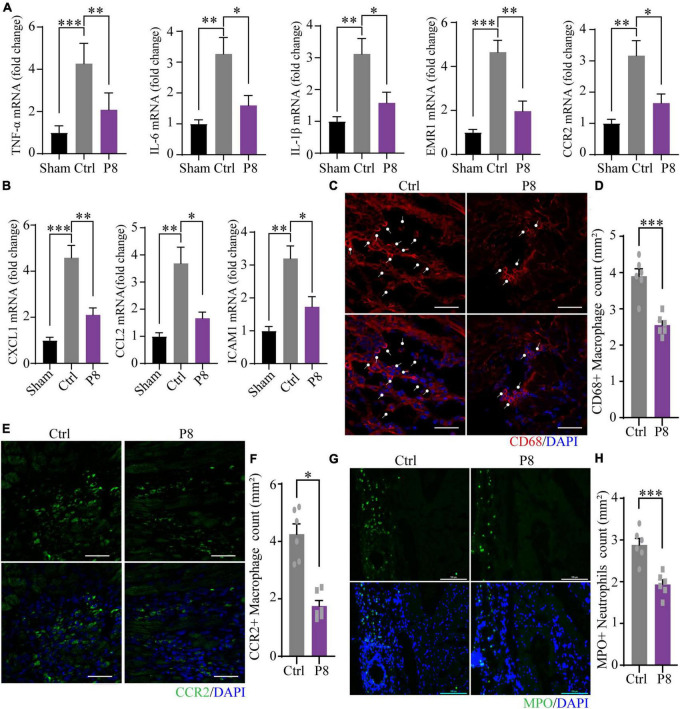
P8 attenuates inflammatory response of heart tissues in the border zone in the infarct border area of mice. **(A)** Real-time PCR analysis of proinflammatory molecules mRNA expressions in border zone of myocardial infarction mice after 3 days with or without P8 administration (*n* = 3 mice). **(B)** mRNA expression of inflammation-related genes (CXCL-1, CCL2, ICAM1 and VCAM1) in border zone of myocardial infarction mice after 3 days with or without P8 administration, as determined with by real-time PCR (*n* = 3 mice). **(C,D)** Immunofluorescence staining and quantitative analyses of heart sections with CD68 antibody. **(E–H)** Representative immunofluorescence images and statistical analysis of with CCR2 **(E,F)** and MPO antibodies **(G,H)** of infarct margin area of heart tissue 3 days after MI. Scale bar: 100 μm. *p < 0.05; **p < 0.01; ***p < 0.001.

To further evaluate the potential mechanism of P8 on NF-κB signaling pathway in mice, we performed RNA sequencing analyses of boarder zone of heart infarction at 3 days after MI. The transcript expression of 178 gene increased and 288 genes decreased in response to P8 treatment (Supplementary Figure 5A). GSEA indicated that TNF signaling pathway and NF-κB signaling pathway were most notably over-represented in the downregulated genes (Supplementary Figures 5A,B). The downregulated expression of proinflammatory genes (CXCL1, ICAM1, TNF-α,IL6, IL-1β,EMR1 and CCR2) and inflammation-related genes (CXCL1, CCL2, ICAM1) was further confirmed by qPCR (Figures 6A,B).

As a key regulator to heart health, macrophages directly promote the formation of collagen and scar in the process of heart repair in MI mice (29). During heart damage, tissue resident and monocyte-derived macrophages were recruited to injured sites and expand. Appropriate inflammation at early stage would benefit to wound healing, whereas extended and excessive inflammation may contribute to collagen and scar formation, thus inhibition of abnormal inflammatory process, such as macrophage homing, has proved to be a promising therapeutic strategy (30). Since P8 decreased the expression of inflammatory genes in the border zone of infarction area, we then examined CD68 + macrophages, pro-inflammatory CCR2 + macrophages and pro-healing CX3CR1 + macrophages, as well as neutrophils. Immunostaining of heart sections at day 3 post-MI showed that compared with the sham controls, CD68^+^ macrophages (Figures 6C,D), pro-inflammatory macrophages (CCR2^+^) (Figures 6E,F) and MPO^+^ neutrophils (Figures 6G,H) significantly decreased. Meanwhile, we observed Ly6G^+^ neutrophils strikingly reduced on day 1 and day 3 post MI after P8 treatment (Supplementary Figures 5C,D). Pro-healing macrophages (CX3CR1^+^) also remarkably increased at day 1 and day 3 post P8 treatment compared with those of the MI controls (Supplementary Figures 5E,F). Through these results, we speculate that P8 may exert its therapeutic effects for ischemic heart damage through inhibiting NF-κB signaling pathway and the regulation of inflammatory cell infiltration.

### Guanosine and deoxynucleoside are the potential effective component of P8

**FIGURE 7:**
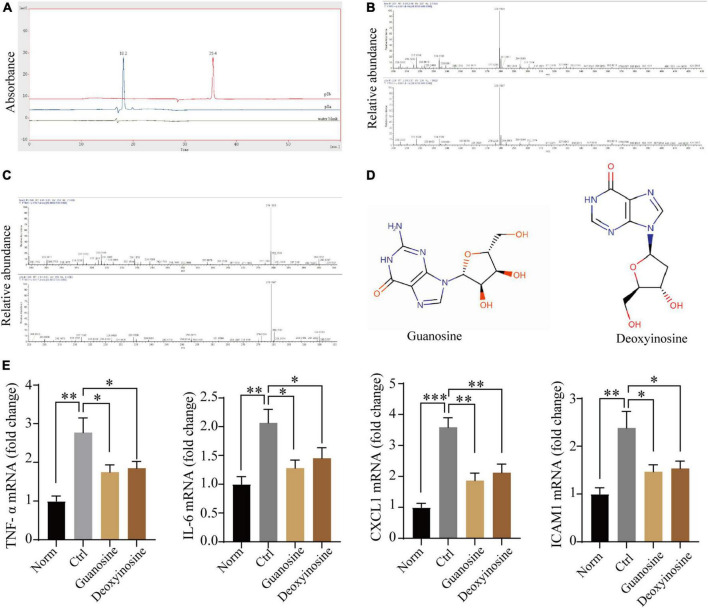
Mass spectrometric analysis of P8. **(A)** P8 showed two main peaks (P8a and P8b) on the Agilent Zorbax AQ column (rt: 12.113;13.627). **(B–D)** Mass spectrometry analysis revealed that guanosine and deoxynucleoside were the main components of Peak 8. **(E)** Transcriptome expression of inflammatory genes in human cardiac cell line AC16 after hypoxia – reoxygenation treatment, with or without Guanosine or deoxyadenosine (μmol/L) by real-time PCR (*n* = 3). *p < 0.05; **p < 0.01; ***p < 0.001.

**FIGURE 8:**
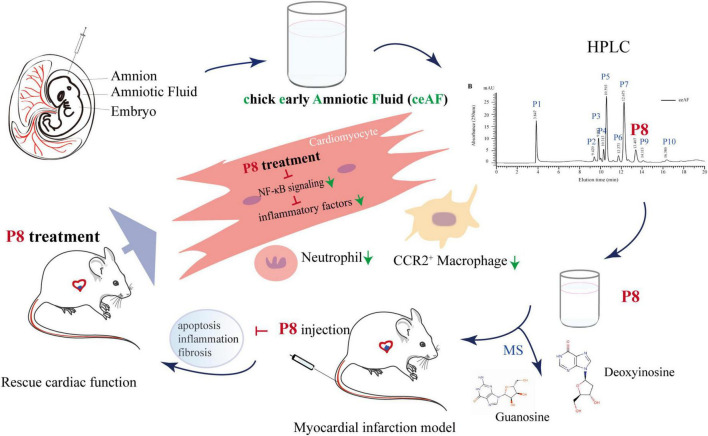
Hypothetical model of P8 in improving heart function in MI mice. ceAF main active component P8 improves cardiac function in MI mice via downregulating NF-κB signaling and inflammation-related gene expression, decreasing infiltration of neutrophils and CCR2^+^ macrophages, and reducing cardiomyocytes apoptosis and cardiac fibrosis.

Finally, we analyzed the components of P8 by mass spectrometry after purification, and we found two main peaks (P8a and P8b) on the Agilent Zorbax AQ column (Figure 7A). Further, standard comparison and analysis revealed that P8a and P8b were guanosine and deoxynucleoside, respectively (Figures 7B–D). Guanosine, an endogenous purine nucleoside is essential for metabolism (31). Recently, it is reported that guanosine has positive inotropic effects in isolated ventricular muscle and atria (32), and alleviates inflammation of airway (33), spinal cord after acute injury (34), brain after traumatic injury (35) and intestinal inflammation (36). Deoxynucleoside can reverse the metabolism of transcriptase inhibitors in perfused heart and isolated mitochondria (37), and had the therapeutic efficacy for Tk2 deficiency (38). In human cardia cell line AC16, guanosine (MedChemExpress, MCE) and deoxyadenosine (MCE) (10 μmol) indeed ameliorated the downregulated inflammatory gene (*TNF*-α, *IL-6*, *CXCL-1* and *ICAM1*) expression induced by hypoxia for 1 h followed by reoxygenation for 2 hs (Figure 7E), suggesting that guanosine and deoxynucleoside may be the potential effective component of P8 in inhibiting inflammatory response. Taken together, our study has demonstrated that ceAF-derived P8 has a heart protection role in MI mice by downregulating inflammatory gene expressions, decreasing the infiltration of inflammatory neutrophils and macrophages, and reducing cardiac apoptosis and fibrosis Figure 8.

## Discussion

Studies about hAF have emerged since Shinya Yamanaka obtained artificial pluripotent stem cells in 2007 when there was an upsurge of regenerative repair research, focusing on the cardioprotective of hAF-derived stem cells after ischemic injury (39, 40). Actually, stem cell therapy largely depends on the acute inflammatory response of macrophages, which can restore the mechanical properties of the infarcted heart (7), while most of administered stem or progenitor cells die within a few days in ischemia-injured animal models (7, 41, 42). Generally, hAF collected during the routine amniocentesis or cesarean section are devoid of any cellular products. Increasing evidences indicate that human amniotic membrane and hAF can alleviate inflammation, have antimicrobial properties, and confer a low risk of immunogenicity (21, 43, 44). The AF of normal human (week 17–20) and rat (E16-17) can improve survival and growth of neuronal and non-neuronal cells as culture media (45). Furthermore, rat AF can induce the formation of duodenal crypts *in vitro* (46), and reduce the ischemia reperfusion damage in rat testes after detorsion (47).

AF is the place where embryos grow, and the composition of AF is distinct in different stages of embryonic development. In fact, unlike humans, chick AF does not collect excretory products and they are swallowed by the embryo to support its growth (48). However, there are few studies on chick embryo AF, partly because the content of early chick AF is low, and the late phase AF contains higher protein content when the albumen transfers from day 12 onward (25). Administration of AF of chick embryo ≥ 11 days by intravenous vein decreases the survival rate in healthy adult C57Bl/6 mice partly due to drastic changes in the AF protein profiles. In the present study, our findings suggest that day 7 AF is safe and P8 component of day 7 AF efficiently improved heart function in mice post-MI.

Our previous study indicated that 5 ml/kg ceAF administration can achieve the same effect as the positive control drug LCZ696, a first line drugs for treatment of heart failure in clinical (49), so in this study 5 ml/kg ceAF was used as the positive control. Although MI mice models are commonly acceptable experiment tool to assess the efficacy of anti-ischemic drugs, differences between human and mouse limited the translational potential of screening new drugs. To overcome the caveats resulted from differences between mice and humans, we further investigated the cardioprotective effects and underlying mechanisms of P8 on acute ischemic injury of heart post-MI and hypoxia/reoxygenation human cell model. With the cell model, P8, the active component of day 7 chick embryo AF, demonstrates anti-hypoxia effects *in vitro* on hESC-derived cardiomyocytes subjected to hypoxia/reoxygenation process. Thus, fewer apoptotic cardiomyocytes appeared in hESC-derived cardiomyocytes subjected to hypoxia/reoxygenation process and the border zone of heart post-MI with P8 treatment. Importantly, P8 has the similar role to ceAF although less effective on improving heart function, suggesting that P8 can be used as a combination drug percutaneous after the surgeries of coronary intervention, thrombolytics, or coronary bypass grafting in clinical.

Microenvironment cues after MI induce inflammatory signals through distinct pathways to furtherly facilitate inflammation and immune cell infiltration. Among these pathways, NF-κB signaling is possibly the best-defined mechanism involved in this process. Interestingly, our data illustrated that P8 downregulated NF-κB signaling pathway, and accordingly reduced the infiltration of pro-inflammatory macrophages and neutrophils in the border zone of heart after MI, which provides an explanation of P8-mediated protective function against MI. Activated NF-κB has also been previously suggested in macrophages (50, 51) and neutrophils (52, 53). We have now revealed a therapeutic role of P8 in suppressing NF-κB pathway in myocardial cells, consolidating blockade of NF-κB pathway after MI might have multifaceted roles. However, our findings cannot exclude the participation of immune cells, particularly macrophages and neutrophils in the protective function of heart by P8. The specific role of P8 in other cells and in-depth mechanisms warrants further investigation.

Notably, we also observed that P8 increases the survival within 1 week after MI model operation in mice, and we speculate that the protective effects of P8 are from reduced fibrosis of cardiomyocytes post-MI. Besides, when harvested the hearts at the end time, we found that P8 attenuates the fibrotic adhesion between the heart and ribs caused by MI model, which was consistent with previous studies that rat AF can reduce the possibility of postoperative intraperitoneal adhesion (20). Adhesion formation after multiple operations is the main cause of infertility, pain, intestinal obstruction, difficulty in reoperation and other complications (20). We envision that chick AF may be administered as a preventive medicine for the topical use during the surgical operations.

## Data availability statement

The original contributions presented in this study are publicly available. This data can be found here: https://www.ncbi.nlm.nih.gov/sra, PRJNA733318 and PRJNA887482.

## Ethics statement

The animal study was reviewed and approved by the Fudan University IACUC, the JOINN Laboratories IACUC (Suzhou, China) and Shanghai University Institutional Animal Care and Use Committee (IACUC).

## Author contributions

BC, XD, NS, and JQ were responsible for the experimental planning, data analysis, and article writing of this study. BC and XC designed and established mice and *in vitro* studies. BC, XC, and YZ performed in mouse and cell studies. BC, XD, and JW wrote and revised the manuscript. LZ performed ceAF extraction and toxicity test. All authors contributed to the article and approved the submitted version.
