# Supplementary material for: Chick early amniotic fluid component improves heart function and protects against inflammation after myocardial infarction in mice
Source: Front Cardiovasc Med. 2022 Nov 16;9:1042852. doi: 10.3389/fcvm.2022.1042852 (PMC9710540; doi:10.3389/fcvm.2022.1042852)
Supplement: Supplementary file 1 [file Data_Sheet_1.docx]

**
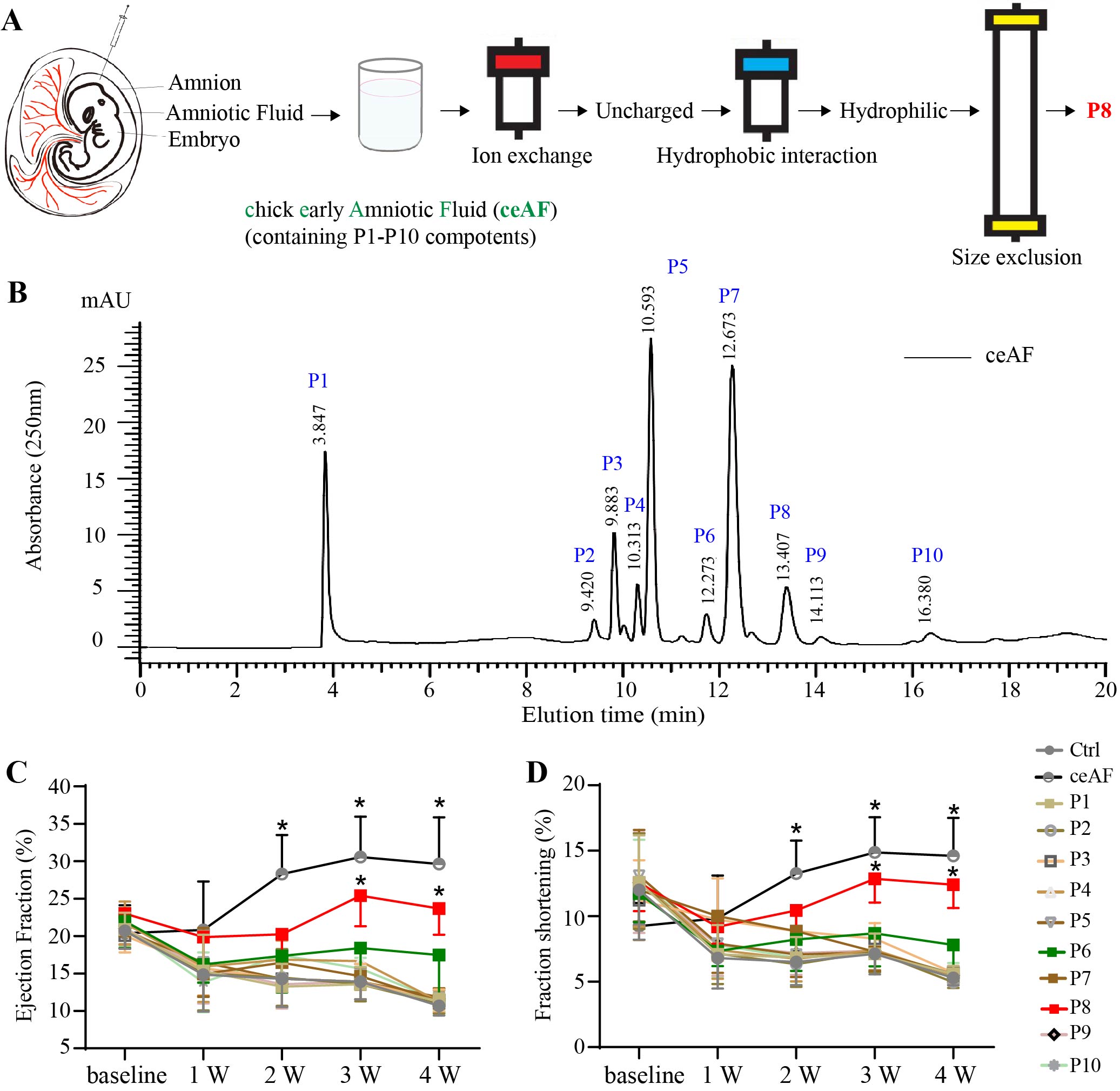
**

**Supplemental Figure 1.** The different peak component extraction of Chick Early Amniotic Fluid (ceAF), quality control through high performance liquid chromatography (HPLC) fingerprint and evaluation of therapy effect on MI mice. **(A)** Schematic diagram of ceAF and different components extraction. (**B**) HPLC chromatogram of the active components of ceAF. Ejection fraction (**C**) and fraction shortening (**D**) were measured after treatment of peak 1-10 components of ceAF in MI mice. The experimental groups are: i.v. injection of 5% glucose (Ctrl) and i.v. injection of ceAF or P 1-10 at 1.5 ml/kg, (n=6).


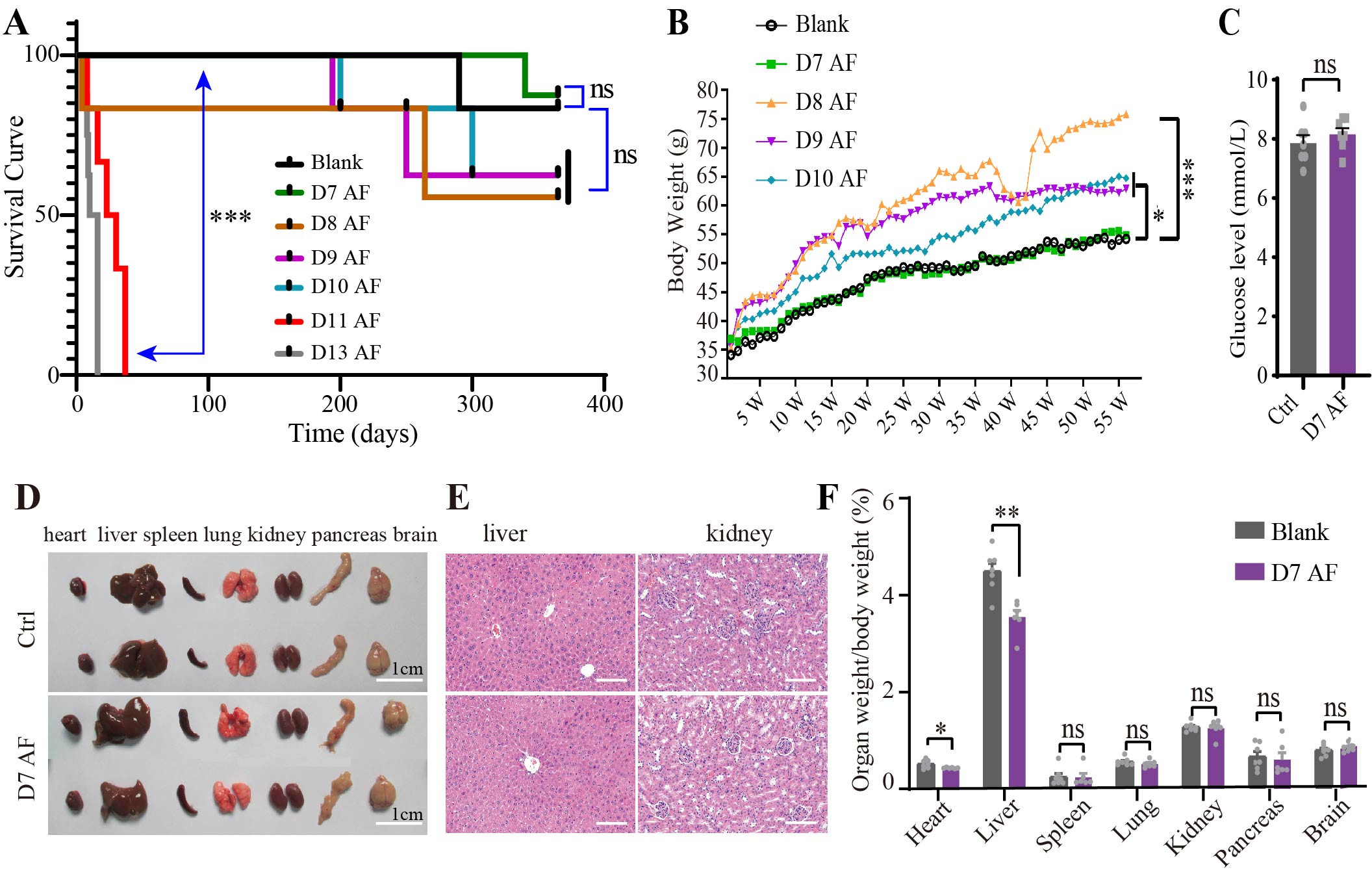


**Supplemental Figure 2.** Long term toxicity test of AF of chick embryo at different time points in mice. (A) The survival rate curve of 7-13 days AF groups and blank control group (5% glucose) at 40 weeks after the first injection. *n* = 14. (B) The average body weight of 7-13 days AF groups and blank control group within 40 weeks after the first injection. (C) The blood glucose of 7-days AF groups and control group was randomly observed after 35 times of injection (day 219), *n* = 6. There was no obvious abnormality in liver and kidney compared with the control group(n=6) from macro images (D, Scale bar: 1cm) and microscopic histopathologic state (Scale bar: 100 μm). (F) The ratio of heart, liver, spleen, lung, kidney, pancreas, brain weight to body weight after 40-weeks D7 AF administration. Data are shown as mean ± SEM. **p* ≤ 0.05, ***p* ≤ 0.01, and ns ≥ 0.05 as determined by one-way ANOVA followed by Tukey’s post hoc test.


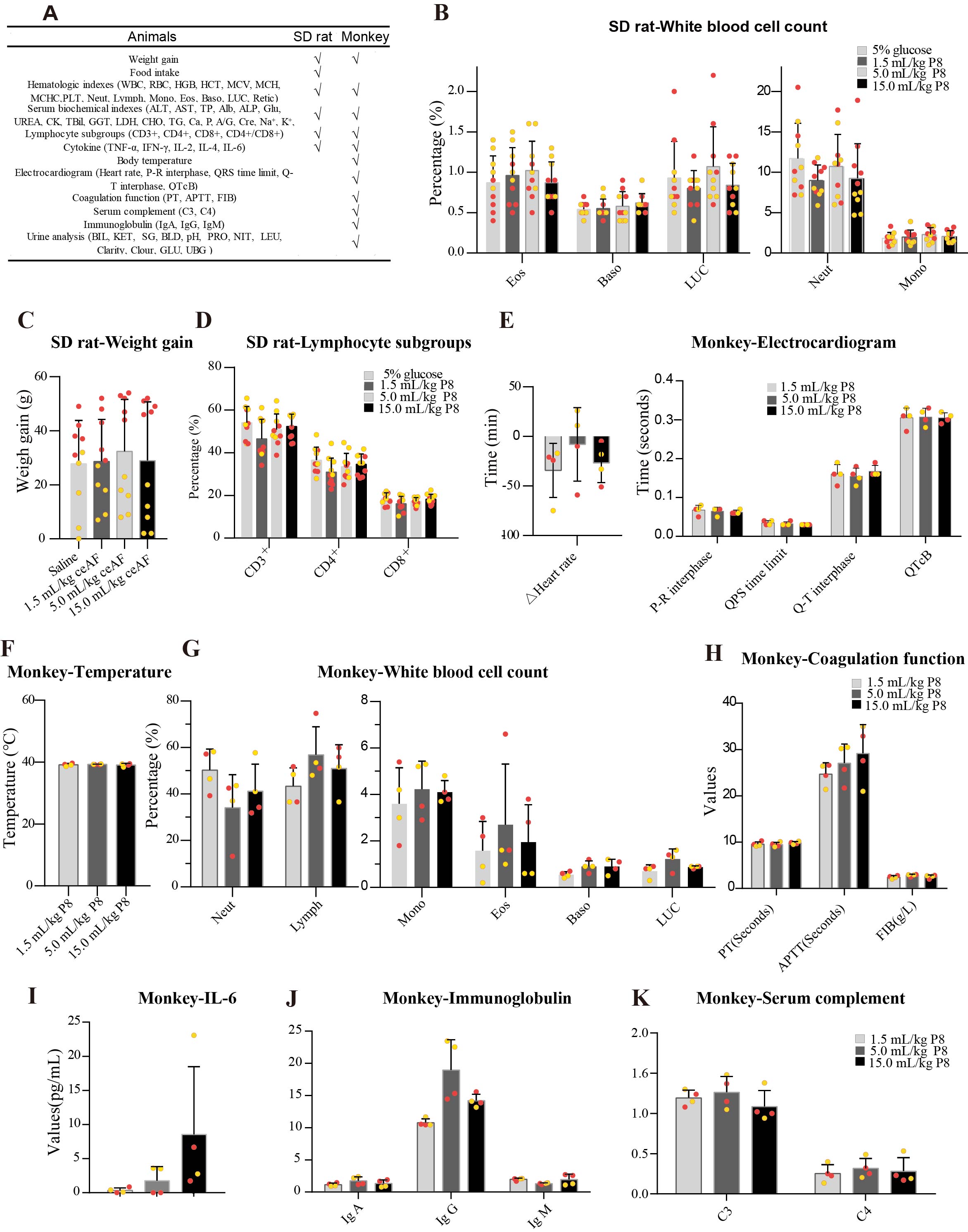


**Supplemental Figure 3** Repeated Intravenous Infusion of P8 for 1 week in SD Rats and Monkeys. (**A)** The summary of toxicological studies by recurring intravenous infusion of P8 in healthy SD rats and monkeys for 1 week. (**B)** The concentrations of cytokines in the serum of the four SD rat groups on the 8th day. (**C)** The lymphocyte subgroup levels in the four SD rat groups on the 8th day. (**D)** The number of white blood cells in the four SD rat groups on the 8th day. (E) The results of electrocardiograms of three groups of monkeys on the 8th day. (**F)** The number of white blood cells in three groups of monkeys on the 8th day. (**G)** The coagulation function of three groups of monkeys on the 8th day. (**H)** The concentrations of cytokines in the serum of three groups of monkeys on the 8th day. (**I**)The concentrations of immunoglobulin in three groups of monkeys on the 8th day. (**J)** The serum complement levels of three groups of monkeys on the 8th day.


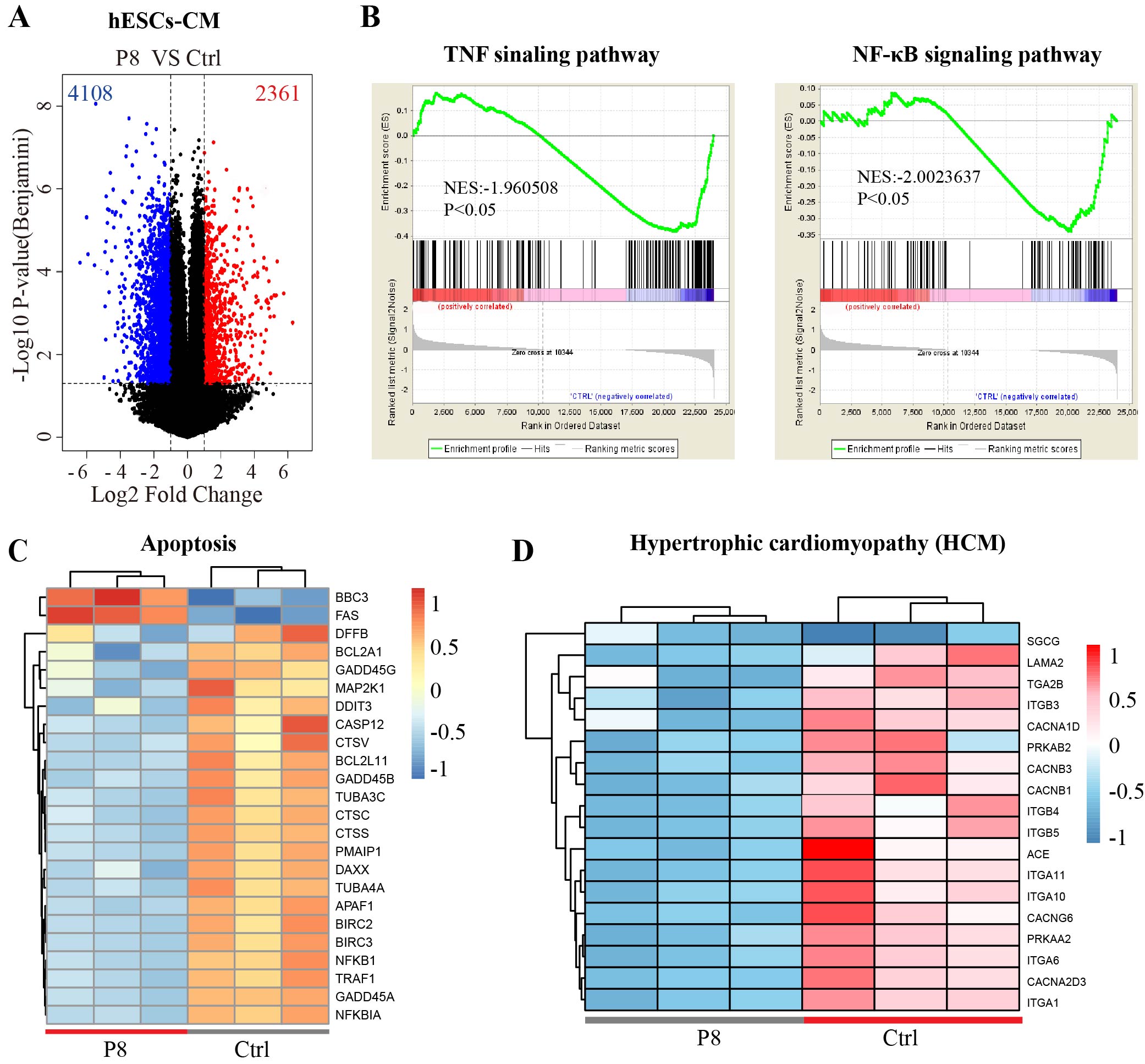


**Supplemental Figure 4** P8 downregulated TNF, NF-κB signaling pathway and the genes of apoptosis and hypertrophic cardiomyopathy (HCM). (**A**) Volcano plots showed that 2361 genes were up-regulated and 4108 genes downregulated in hESCs-derived cardiomyocytes subjected to hypoxia in a hypoxic chamber (1% O_2_, 5% CO_2_, and 94% N_2_) for 2 hours followed by 1 hour of reoxygenation with or without P8 treatment (*n* = 3 per group). (**B**) Pathways downregulated in hESCs-derived cardiomyocytes subjected to hypoxia in a hypoxic chamber (1% O_2_, 5% CO_2_, and 94% N_2_) for 2 hours followed by 1 hour of reoxygenation with treatment (*n* = 3 per group) compared with control with GSEA analysis by choosing Hallmarks as the gene set correlation. The apoptosis-(**C**) and HCM-related (**D**) genes are shown in heatmaps.


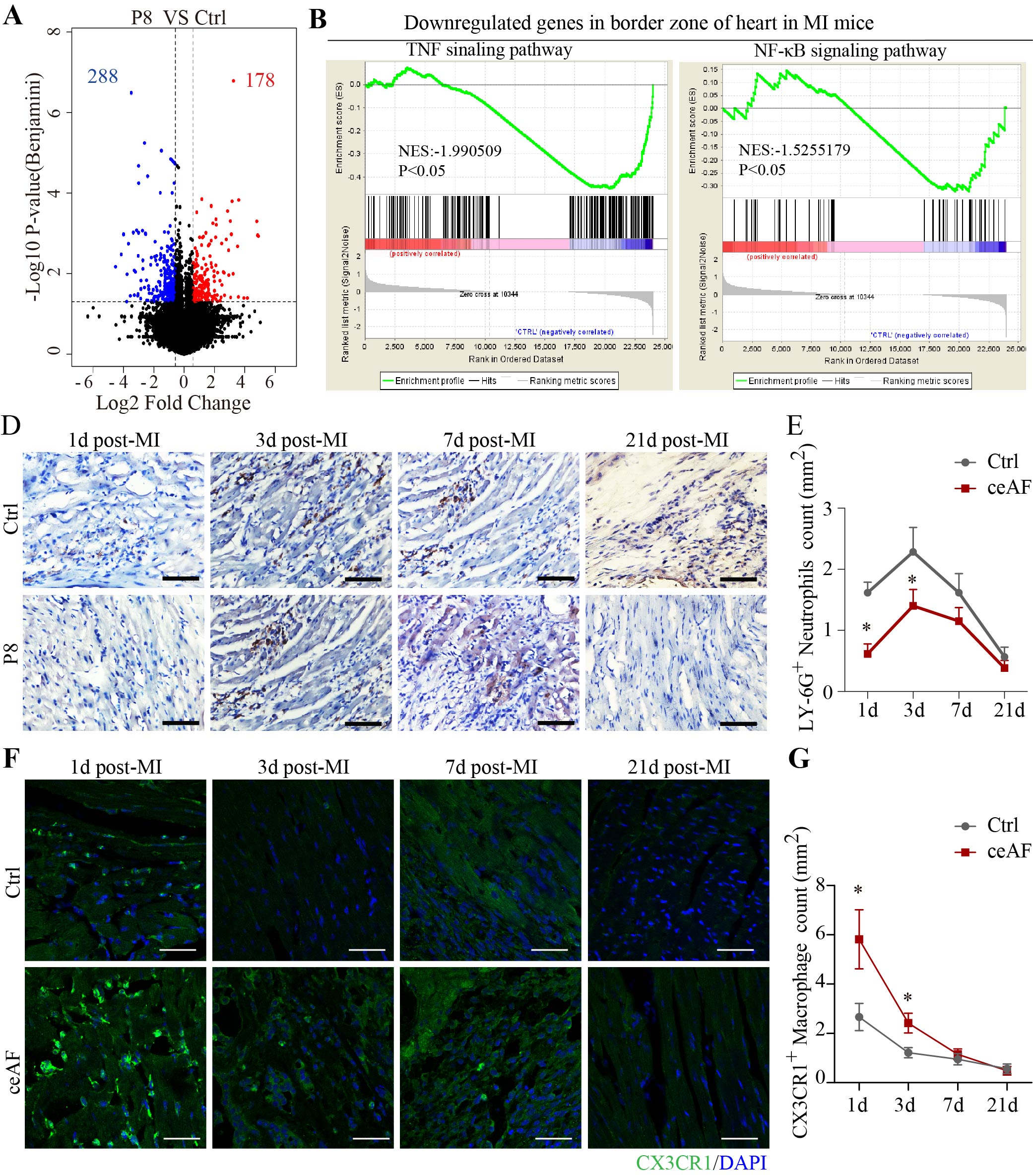


**Supplemental Figure 5** P8 altered inflammatory responses of heart tissues in the border zone of infarction after MI. (**A**) Volcano plots of the RNA-seq data of P8- treated group versus control group with 5% glucose administration at 3 days after MI (*n* = 3 per group) showed that 178 genes were up-regulated and 288 genes downregulated. (**B**) Pathways downregulated in myocardial infarcted area of P8-treated mice (*n* = 3) compared with Glucose-treated mice with GSEA analysis by choosing Hallmarks as the gene set correlation. All pathways with GSEA *P* < 0.05 were shown. (**C-D)** Representative images and quantitative analyses of immunofluorescence staining with anti-CCR2 (green) of heart tissues in the border zone of infarction 1 day, 3 days, 7 days, and 21 days after MI. (**E-F)** Representative images and quantitative analyses of immunohistochemistry with anti-Ly-6G (brown) of heart tissues in the border zone of infarction 1 day, 3 days, 7 days, and 21 days after MI. Scale bar: 100 μm. *, *p* < 0.05; *.
