# Supplementary material for: Chick early amniotic fluid component improves heart function and protects against inflammation after myocardial infarction in mice
Source: Front Cardiovasc Med. 2022 Nov 16;9:1042852. doi: 10.3389/fcvm.2022.1042852 (PMC9710540; doi:10.3389/fcvm.2022.1042852)
Supplement: Supplementary file 2 [file Table_1.docx]

Supplementary Table 1. qPCR Primers for hESC-derived CMs

| Primer | Forward | Reverse |
| --- | --- | --- |
| GAPDH | GGCAAATTCCATGGCACCG | ATGACGAACATGGGGGCATC |
| TNF-α | GCTGCACTTTGGAGTGATCG | TATCTCTCAGCTCCACGCCA |
| IL1β | ACCTGAGCTCGCCAGTGAAA | AAGGTGCTCAGGTCATTCTCC |
| IL6 | GCAAAGAGGCACTGGCAGAA | TTGGGTCAGGGGTGGTTATTG |
| EMR1 | CCTCCAGCACATCCAGCCAAA | GGCGAGACATACCAGAGAGATG |
| CCR2 | TGGTCATCTGCTACTCGGGA | TGTGAAAAAGGCTTCTGAACTTCT |
| CXCL1 | CAGCAGGAGCGTCCGT | GATTTGTCACTGTTCAGCATCTTT |
| ICAM1 | CGACTGGACGAGAGGGATTG | GGAGAGCACATTCACGGTCA |
