# Supplementary material for: Chick early amniotic fluid component improves heart function and protects against inflammation after myocardial infarction in mice
Source: Front Cardiovasc Med. 2022 Nov 16;9:1042852. doi: 10.3389/fcvm.2022.1042852 (PMC9710540; doi:10.3389/fcvm.2022.1042852)
Supplement: Supplementary file 3 [file Table_2.DOCX]

Supplementary Table 2. qPCR Primers for mouse

| Primer | Forward | Reverse |
| --- | --- | --- |
| GAPDH | AGGTCGGTGTGAACGGATTTG | TGTAGACCATGTAGTTGAGGTCA |
| TNF-α | CCCTCACACTCAGATCATCTTCT | GCTACGACGTGGGCTACAG |
| IL1β | GCAACTGTTCCTGAACTCAACT | ATCTTTTGGGGTCCGTCAACT |
| IL6 | TAGTCCTTCCTACCCCAATTTCC | TTGGTCCTTAGCCACTCCTTC |
| EMR1 | CCTCCAGCACATCCAGCCAAA | GGCGAGACATACCAGAGAGATG |
| CCR2 | GCCACCACACCGTATGACTA | GCAGCATAGTGAGCCCAGAA |
| CXCL1 | CCTATCGCCAATGAGCTGC | ACTTGGGGACACCTTTTAGC |
| ICAM1 | TTTTGGAGCTAGCGGACCAG | AAACAGGAACTTTCCCGCCA |
| CCL2 | CACTCACCTGCTGCTACTCA | AGACCTTAGGGCAGATGCAG |
| ANP | ATCCTGTGTACAGTGCGGTG | GATCTATCGGAGGGGTCCCA |
| BNP | AAAGTCGGAGGAAATGGCCC | TTCAGTGCGTTACAGCCCAA |
